# Influence of parental smoking on the use of alcohol and illicit drugs among adolescents

**DOI:** 10.31744/einstein_journal/2019AO4377

**Published:** 2018-12-28

**Authors:** Luciano Machado Ferreira Tenório de Oliveira, Ana Raquel Mendes dos Santos, Breno Quintella Farah, Raphael Mendes Ritti-Dias, Clara Maria Silvestre Monteiro de Freitas, Paula Rejane Beserra Diniz

**Affiliations:** 1 Centro Universitário Boa Viagem, Recife, PE, Brazil; Centro Universitário Tabosa de Almeida, Caruaru, PE, Brazil; Centro Universitário Tabosa de Almeida, Caruaru, PE, Brazil; 2 Universidade de Pernambuco, Recife, PE, Brazil; 3 Universidade Federal Rural de Pernambuco, Recife, PE, Brazil; 4 Universidade Nove de Julho, São Paulo, SP, Brazil; 5 Universidade Federal de Pernambuco, Recife, PE, Brazil

**Keywords:** Tobacco use disorder, Street drugs, Adolescent, Public Health, Parents, Parenting, Tabagismo, Drogas ilícitas, Adolescente, Saúde Pública, Pais, Poder familiar

## Abstract

**Objective:**

To evaluate the association between parental smoking and the use of alcohol and illicit drugs among adolescent children.

**Methods:**

A cross-sectional study with 6,264 adolescents (59.7% female) aged between 14 and 19 years. To establish the sample, we used two-stage cluster random sampling. The data on parental smoking and use of cigarettes, alcohol and illicit drugs among adolescents were collected using a questionnaire.

**Results:**

Smoking adolescents were more prone to use alcohol (odds ratio − OR: 10.35; 95%CI: 7.85-13.65) and illicit drugs (OR: 11.75; 95%CI: 9.04-15.26) than non-smokers (p<0.001). Adolescents with at least one parent (OR: 1.4; 95%CI: 1.13-1.89) or both parents smoking (OR: 1.6; 95%CI: 1.01-2.67) were more likely to smoke when compared to those having no parents smoking. The adjusted analysis limited to non-smoking adolescents showed a positive association (p<0.05) between parental tobacco use and the use of alcohol (OR: 1.4; 95%CI: 1.23-1.62) and illicit drugs (OR: 1.6; 95%CI: 1.24-2.13), irrespective of age, sex, maternal schooling and place of residence.

**Conclusion:**

Parental smoking was associated with the use of alcohol and other illicit drugs by adolescents, even among nonsmokers.

## RESUMO

**Objetivo:**

Analisar a associação entre tabagismo parental e uso de álcool e drogas ilícitas dos filhos adolescentes.

**Métodos:**

Estudo transversal conduzido com 6.264 adolescentes (59,7% meninas) com idade entre 14 e 19 anos. Para estabelecimento da amostra, recorreu-se à amostragem aleatória por conglomerados em dois estágios. Os dados sobre tabagismo dos pais e uso de cigarros, álcool e drogas ilícitas entre os adolescentes foram obtidos por questionário.

**Resultados:**

Os adolescentes fumantes eram mais propensos a usar álcool (*odds ratio* − OR: 10,35; IC95%: 7,85-13,65) e drogas ilícitas (OR: 11,75; IC95%: 9,04-15,26) do que os não fumantes (p<0,001). Os adolescentes que pelo menos um dos pais fumava (OR: 1,4; IC95%: 1,13-1,89) ou os dois fumavam (OR: 1,6; IC95%: 1,01-2,67) tiveram mais chances de fumar quando comparados aos que não tinham pais fumantes. Análise ajustada limitada a adolescentes não fumantes revelou associação positiva (p<0,05) entre o tabagismo dos pais e o consumo de álcool (OR: 1,4; IC95%: 1,23-1,62) e drogas ilícitas (OR: 1,6; IC95%: 1,24-2,13), independentemente de sexo, idade, escolaridade materna e região de moradia.

**Conclusão:**

O tabagismo dos pais esteve associado com o consumo de álcool e outras drogas ilícitas em adolescentes, até mesmo entre os não fumantes.

## INTRODUCTION

Adolescence is a period of development characterized by several biological, psychological and social changes, which may predispose youth to some risk behaviors, such as the use of illicit drugs.^(^ ^1^ ^,^ ^2^ ^)^ One of the factors related to smoking in adolescence is parental tobacco use.^(^ ^3^ ^,^ ^4^ ^)^ Another concern is that smoking adolescents are more prone to use illicit drugs, alcohol,^(^ ^5^ ^,^ ^6^ ^)^ or both.^(^ ^7^ ^)^

Despite the well established relation between tobacco use by parents and by children,^(^ ^3^ ^,^ ^4^ ^)^ no association has been proven so far between parental smoking and the use of alcohol and illicit drugs by their children. Neither if this relation would also be applicable to non-smoking adolescents, considering that tobacco use by youth is associated with the use of illicit drugs^(^ ^6^ ^,^ ^8^ ^)^ and alcohol;^(^ ^5^ ^,^ ^6^ ^)^ therefore, these behaviors are considered confounding factors and variables that must be controlled.

It should be noted that the use of cigarettes, alcohol and illicit drugs may start during childhood,^(^ ^9^ ^,^ ^10^ ^)^ and it is essential to monitor factors which may increase the risk for initiation and, therefore, the development of diseases related with said behaviors, which are directly associated with higher morbidity and mortality among adolescents.^(^ ^11^ ^)^

## OBJECTIVE

To evaluate the association between parental smoking and the use of alcohol and illicit drugs among adolescent children.

## METHODS

A cross-sectional, school-based, state wide, quantitative research study using data from the project called *“Physical activity and health-risk behaviors among high-school students of the state of Pernambuco”.* One of the specific objectives of this project was to “define the subgroups of the study target population with the highest chance of exposing themselves to health-risk behaviors.

The study was conducted in 2011 with students enrolled in public high schools of the state of Pernambuco, aged between 14 and 19 years. The study was approved by the Institutional Review Board of the *Universidade do Pernambuc*o (CAAE: 0158.0.097.000-10CEP-UPE: 159/10).

To ensure that the sample selected was representative of the target population, the size of the schools was considered: schools with less than 200 students were considered small; with 200 to 499 students, medium; and with more than 500 students, large. The distribution of students between day and night classes was also considered. Students attending morning and afternoon classes were placed into one single category (daytime students). The regional distribution factored in the number of schools in each of the 17 Regional Education Offices.

The study used stratified, two-stage cluster random sampling. The school and the classroom represented the sampling units for the first and second stages, respectively. All schools in the state public education network offering regular, high school education were considered eligible for enrollment in the study. In the first stage, the stratification criterion adopted was the density of schools in each of the state's micro-regions, according to their size, and more schools were proportionally selected in the micro-regions with greater density. In the second stage, the density of classrooms in the schools, randomly selected by period (daytime and nighttime), was considered as the criterion to randomly determine in which schools the questionnaires would be applied.

The schools and classrooms were randomized using the electronic platform https://www.randomizer.org/, which provided random numbers. All students in the classrooms selected were invited to take part in the study, regardless of their age. The inclusion criterion adopted was adolescents regularly enrolled in public high schools of the state of Pernambuco. The exclusion criteria included inappropriate filling of the questionnaires, adolescents aged under 14 and over 19, students who were absent on the day the questionnaire was applied, or students and/or guardians refusing to take part in the research study.

To calculate the sample size, the following parameters were used: 95% confidence interval (95%CI), maximum tolerable error of two percentage points; design effect (deff) = 2; and, since the study comprised the analysis of multiple risk behaviors with different frequencies of occurrence, the estimated prevalence was defined at 50%.

The data collection used a translated and adapted version of the Global School-based Student Health Survey, previously used with adolescents.^(^ ^12^ ^)^ It took place between May and November 2011, and the questionnaire was applied in group interviews.

A pilot study was conducted to test the applicability of the questionnaire. The data in this pilot study were collected from a reference state school of the public education network in the city of Recife, with a sample of 86 adolescents aged between 14 and 19 years. Reproducibility indicators had moderate to high intraclass correlation coefficients for most questionnaire items, and all concordance correlation coefficients (Kappa index) varied between 0.52 and 1.00. The time for completion of the questionnaire was approximately 40 to 50 minutes.

Personal information, socioeconomic and sociodemographic variables were obtained through direct questions about sex, age, skin color, marital status, place of residence, occupation and maternal level of schooling, such as: “What is you sex?”; “How old are you, in years?”, “Do you consider yourself: white, black, brown, yellow or Indian?”; “What is your marital status?; “Is your residence located in an urban or rural region/area?”; “Do you work?”; and “Which best indicates your mother's level of schooling?”, respectively.

The outcome variables analyzed were the use of alcohol, illicit drugs and cigarettes. Alcohol use was assessed by the question: “in the last 30 days, on how many days did you drink at least one dose of an alcohol-containing beverage?”. Tobacco use was assessed by the question: “in the last 30 days, on how many days did you smoke cigarettes?”. Adolescents who reported having used alcohol, tobacco or illicit drugs at least once in the last 30 days were considered exposed.^(^ ^13^ ^)^ The use of illicit drugs was assessed by the question: “in your lifetime, how many times have you used drugs such as “*loló*” (ether-based spray), shoemaker's glue, marijuana, crack, cocaine or others (not including cigarettes or alcohol)?”. Adolescents who reported using any of these substances in their lifetime were considered exposed.^(^ ^14^ ^)^

The predictor variable considered in this study was parental tobacco use, evaluated by the adolescents’ answer to the question “Which of your parents or guardians use any form of tobacco?”, categorized as “neither of them smokes”, “one of them smokes” and “both of them smoke”. The use of tobacco by the adolescents was considered a potential confounding factor, in addition to demographic factors, like sex, age, place of residence and maternal schooling.

The tabulation process was carried out in the EpiData software, version 3.1, using electronic procedures for data entry control, with the check functionality. We resorted to double-entry verification to ensure consistent data entry. All data entry typos were identified and corrected. The data analysis was made using the Statistical Package for Social Science (SPSS), version 10.0, for Windows.

The data analysis included descriptive statistics (frequency distribution) and association measurements (Pearson's χ^2^ and binary logistic regression). Binary logistic regressions were used to assess the association between parental tobacco use and the use of cigarettes, alcohol and illicit drugs, with control of the variables sex, age, maternal schooling and place of residence. The criterion for entry of variables in the statistical model was p<0.20, using the backward method. The results are demonstrated by an estimation of the odds ratio (OR) and the 95%CI.

All subjects signed an Informed Consent Form. Students aged under 18 years, on the day of their first school visit, received a Negative Consent Form to be given to their parents or guardians. If the parents/guardians did not consent to their child participating in the study, the document would have to be handed in on the day scheduled for data collection, completed (with the child's name and the parent's name, signature and contact telephone number), with a check on the option stating “I do not authorize my child to take part in this study”. The questionnaires did not display any form of personal identification, to ensure the confidentiality of the answers.

## RESULTS

In 48 cities of the State of Pernambuco, 85 schools were visited and 7,195 adolescents were evaluated, of which 919 were excluded for being aged <13 or >19 years. In addition, 15 adolescents were excluded for not completing the questionnaire. Thus, the sample of this study was 6,264 adolescents (59.7% female) and the mean age was 16.6±1.2 years. Sample characteristics and the prevalence of risk behaviors are presented in table 1.

**Table 1:** Demographic characteristics and health indicators of adolescents*

Variables		Results
Sex (n=6,261)		
	Male	2,524 (40.3)
	Female	3,737 (59.7)
Age, years (n=6,264)		
	14-15	1,350 (21.6)
	16-17	3,345 (53.4)
	18-19	1,569 (25.0)
Place of residence (n=6,234)		
	Urban	4,646 (74.5)
	Rural	1,588 (25.5)
Maternal level of education, years of schooling (n=5,394)		
	>8	1,903 (35.3)
	≤8	3,491 (64.7)
Parental smoking (n=6,081)		
	No	4,407 (72.5)
	Yes	1,674 (27.5)
Cigarettes (n=6,251)		
	No	5,926 (94.8)
	Yes	325 (5.2)
Alcohol (n=6,257)		
	No	4,467 (71.4)
	Yes	1,790 (28.6)
Illicit drugs (n=6,258)		
	No	5,843 (93.4)
	Yes	415 (6.6)

Results expressed by n (%).

* due to unanswered questions, the number of subjects varied when comparing the different variables; ^†^ “*loló*” (ether-based spray), shoemaker's glue, “*lança-perfume*” (another name for “*loló*”), marijuana, crack, cocaine or others (not including cigarettes or alcohol).

After controlling for sex, age, maternal schooling and place of residence, we observed that smoking adolescents had a higher chance of using alcohol (OR: 10.35; 95%CI: 7.85-13.65) and illicit drugs (OR: 11.75; 95%CI: 9.04-15.26) than non-smokers, as shown in table 2.

**Table 2:** Association between smoking and the use of alcohol and illicit drugs among adolescents (n=6,264)

		Gross OR	95%CI	Adjusted OR*	95%CI
Alcohol					
	No	1		1	
	Yes	11.3†	8.5-14.9	10.2†	7.60-13.7
Illicit drugs‡					
	No	1		1	
	Yes	12.8†	9.9-16.5	11.7†	8.86-15.4

* adjusted for sex, age, maternal schooling and place of residence; p value<0.05;

‡ “*loló*” (ether-based spray), shoemaker's glue, “*lança-perfume* ” (another name for “*loló*”), marijuana, crack, cocaine or others (not including cigarettes or alcohol).

OR: odds ratio; 95%CI: 95% confidence interval.

**Figure 1:**
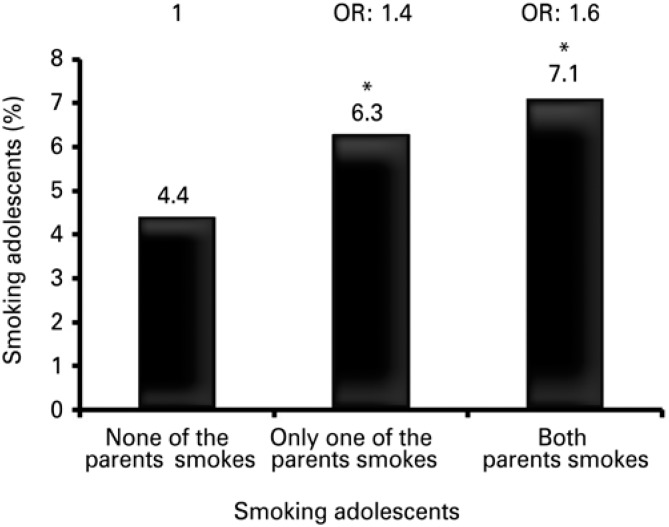
Prevalence of smoking among adolescents in relation to parental smoking * p value<0.05. OR: odds ratio.

Adolescents with at least one parent smoking (OR: 1.4; 95%CI: 1.13-1.89) or both parents smoking (OR: 1.6; 95%CI: 1.01-2.67) had a higher chance of smoking when compared to those with neither parent smoking (Figure 1).

The adjusted analyses (sex, age, maternal schooling, place of residence and adolescent smoking) showed a positive association between parental tobacco use and the use of alcohol (OR: 1.4; 95%CI: 1.3-1.6) and illicit drugs (OR: 1.6; 95%CI: 1.2-2.0), even among non-smoking adolescents (Table 3).

**Table 3:** Association between parental smoking and the use of alcohol and illicit drugs among non-smoking adolescents

		All adolescents (n=6,264)		Non-smoking adolescents (n=5,926)	
		Adjusted OR*	95%CI	Adjusted OR*	95%CI
Tobacco					
	No	1			
	Yes	1.5†	1.2-2.0		
Alcohol					
	No	1		1	
	Yes	1.4‡	1.3-1.6	1,4‡	1.2-1.6
Illicit drugs§					
	No	1		1	
	Yes	1.6†	1.2-2.0	1.6‡	1.2-2.1

* adjusted for sex, age, maternal schooling and place of residence;

† p value<0.05;

‡ p value<0.001;

§ “*loló*” (ether-based spray), shoemaker's glue, *lança-perfume* (another name for “*loló*”), marijuana, crack, cocaine or others (not including cigarettes or alcohol).

OR: odds ratio; 95%CI: 95% confidence interval.

## DISCUSSION

The prevalence of unhealthy behaviors, such as the use of cigarettes, alcohol and illicit drugs, in this study sample, is similar to that found in investigations from other countries.^(^ ^15^ ^–^ ^19^ ^)^ Even more concerning is the fact that, when transitioning from childhood to adulthood, many adolescents get involved with multiple risk behaviors which may persist in adult life,^(^ ^20^ ^)^ and the onset of these behaviors during adolescence represents a Public Health concern. These risk behaviors are also directly associated with higher adolescent morbidity and mortality rates.^(^ ^11^ ^)^

As previously noted,^(^ ^8^ ^,^ ^21^ ^)^ this study found that adolescents who smoked where more likely to use illicit drugs and alcohol. Although the cross-sectional design of the study can limit its ability to explain the phenomenon as a whole, one may speculate that tobacco use may encourage adolescents to use other more hazardous drugs, particularly during adolescence, which is a period characterized by new discoveries and conflicts.^(^ ^22^ ^)^ The use of these substances has a direct impact on adolescents´ health, and is directly associated with mortality from external causes.^(^ ^10^ ^)^

Another interesting finding of this study was that adolescents with smoking parents were more likely to use tobacco, alcohol and illicit drugs. Smoking cigarettes is an important predictor of the use of other illicit drugs,^(^ ^23^ ^)^ and parental risk behaviors are associated with similar behaviors among their adolescent children.^(^ ^24^ ^)^Abreu et al.,^(^ ^25^ ^)^ for instance, observed that 12.8% of Brazilian youth smoke cigarettes, and demonstrated the influence of family or friends is the main causal factor to start smoking. Another study with 658 adolescents aged between 14 and 17 showed that parents’ awareness of their children's habits, having household rules enforced, and the explicit disapproval of cigarette smoking may discourage adolescents from starting smoking.^(^ ^26^ ^)^

Another aspect observed in this study, which corroborates the influence of parental smoking on health-related behaviors of their children is the fact that adolescents with smoking parents were more likely to use alcohol and illicit drugs, irrespective of their own smoking habits. In support of this finding, Taylor et al.,^(^ ^27^ ^)^ stated that the social environment (parents, siblings and friends) can strongly affect the chance of adolescents using alcohol. Licanin^(^ ^28^ ^)^ found 39.4% of alcohol use and 2.2% of marijuana use among adolescents with smoking parents.

In addition to imitation of parental habits by their children, another point worth noting is the potential genetic predisposition to chemical dependence, in which alcohol, illicit drugs and tobacco dependence can be passed from parent to child.^(^ ^29^ ^)^ For this reason, one of the ways to reduce tobacco use among adolescents is getting parents to quit smoking.^(^ ^30^ ^)^

This study has limitations and strengths that must be considered. Among the limitations, we highlight the cross-sectional design of this study, which did not allow us to establish a causal relationship between the outcome and independent variables. Another limitation was the non-control of the use of illicit drugs and alcohol by parents, which can represent a major confounder, which is frequently not controlled. The strengths of this study include the size and representativeness of the sample, which included adolescents from all over the state of Pernambuco, as well as control for demographic variables, which are major confounding factors.

## CONCLUSION

This study revealed, in a representative sample of students, that parental tobacco use was associated with the use of alcohol and illicit drugs by their children, even among non-smoking adolescents.
